# RNA: a possible contributor to the 'missing heritability’

**DOI:** 10.1186/2051-4190-23-9

**Published:** 2013-11-01

**Authors:** Valérie Grandjean, Danielle A Badro, Jafar Kiani

**Affiliations:** University of Nice, INSERM U636, Parc Valrose, Nice, 06100 France

**Keywords:** ARN non-codant, Épigénétique, Hérédité, Gamète, Non-coding RNA, Inheritance, Epigenetics

## Abstract

A number of human pathologies have a transmission pattern that does not obey Mendelian segregation rules. This type of heredity is defined as non-Mendelian and is based on mechanisms of transgenerational epigenetic inheritance. Comprehensive information on the molecular mechanisms of it is still lacking. However, recent evidence from distantly related species including *Caenorhabditis elegans*, *Drosophila*, and mouse, points towards a role for non-coding RNA molecules in such a pattern of inheritance. While it would be too hasty to conclude that RNA molecules are at work in the transgenerational non-genetic inheritance of human pathologies, a growing number of studies seem to strongly support such a speculation.

## Résumé

De nombreuses études scientifiques ont clairement établi que nos différentes expériences de vie, notamment notre alimentation, ont un impact crucial sur l’expression de nos gènes, ainsi que sur celle de notre descendance. Le plus souvent, la transmission de telle maladie et/ou de tel phénotype nouvellement acquis ne suit pas les lois de Mendel et est souvent associée à des altérations non génétiques. Cette hérédité, dite hérédité épigénétique, est encore très mal connue sur le plan moléculaire. Néanmoins, de nombreuses analyses réalisées sur des organismes très éloignés sur le plan évolutif, allant du nématode *C. elegans* à la souris, en passant par la mouche Drosophile, montrent que les molécules d’ARN fournies par le gamète à l’embryon y joueraient un rôle déterminant. Bien qu’il soit prématuré de confirmer que les molécules d’ARN pourraient jouer le même rôle dans la transmission de certaines pathologies humaines, l’ensemble de ces données tend à montrer que cela pourrait bien être le cas.

## Introduction

How biological systems respond and adapt to rapid environmental changes is a challenging question. Over the past few years, a growing body of evidence has involved epigenetic (non-genetic) modifications in this adaptation process. By contrast to genetic information, epigenetic information is not systematically fixed and can be either dynamic or stable. The dynamics of epigenetic marks make it possible to respond reversibly to environmental signals, but also to firmly stabilize cell-type-specific gene programs. Thus, as a consequence of specific conditions such as a new environmental stress (prenatal and early postnatal exposure to environmental factors) and/or a genetic mutation, a new epigenetic profile can be established, maintained in the germ line, and propagated to the following generations. This process has been defined as “epigenetic transgenerational inheritance” [1]. During mammalian development, there are mainly two phases when new epigenetic profiles are entirely reprogrammed. The first phase occurs during gametogenesis when primordial germ cells enter the gonads in mid-gestation and start adopting sex-specific fates. The second occurs in the zygote and correlates with removal of sperm- and oocyte-specific epigenetic programs, and reactivation of the totipotency. These windows of reprogramming are crucial and may represent the critical developmental time points for the introduction of epigenetic changes or potential errors through exposure to environmental factors [2, 3]. Until now, DNA methylation and chromatin modifications have been considered as an interface between environmental factors and genetic information in the living organisms. However, recent evidence points towards a role for diffusible factors, in particular RNA molecules. This review focuses on this challenging aspect of RNA-mediated epigenetic transgenerational inheritance. The potential role of non coding RNAs, especially sperm RNAs, will be discussed with regard to evidence of transgenerational epigenetic inheritance of acquired phenotypes induced by environmental changes and RNA-mediated transgenerational inheritance of new regulatory states.

### Evidence of non-genetic inheritance of newly acquired phenotypes induced by environmental changes

#### ***Nutritional effects and health consequences on the next generation***

A number of recent studies have drawn attention to the role of the paternal diet on the development of complex diseases such as obesity and type 2 diabetes. This was, in particular, suggested by the epidemiological Överkalix Cohort Study conducted on residents of an isolated community in the far northeast of Sweden [4, 5]. The authors found that poor diet during the paternal grandfather’s slow growth period (i.e., before puberty, between the age of 9 and 12), increased the risk of diabetes, obesity, and cardiovascular diseases in the second-generation of offspring. Studies on the molecular mechanisms involved in such type of inheritance in humans have been hindered by factors such as social, environmental, and economical variables, and have therefore triggered the generation of rodent models.

Ng and colleagues fed male rats with a high-fat diet and looked for effects in their adult female offspring which were fed with a normal diet. These daughters exhibited early-onset of impaired insulin secretion, glucose tolerance, and normal adiposity. This was associated with altered expression levels of 642 pancreatic islet genes. Interestingly, hypomethylation at the interleukin-13 receptor-α2 (*Il13ra2*) locus was associated with the largest alteration in expression level [6].

In another study, Carone and colleagues fed male mice with a low-protein diet [7]. Expression profile screening of genes in offspring of both sexes revealed elevated hepatic expression of many genes involved in lipid and cholesterol biosynthesis. Modest changes in DNA methylation were detected at many sites, including a consistent change close to the *Ppara* gene encoding the peroxisome proliferator-activated receptor-α, a regulator of lipid metabolism. Although molecular analyses indicated that DNA methylation might be involved in this type of regulation, the implication of other mechanisms cannot be excluded.

In brief, these studies provide evidence that diet can heritably influence gene expression patterns by modifying epigenetic profiles. Such an association between diet and epigenome might be an underlying factor in the epidemic levels of obesity and metabolic syndromes that have become major public health problems in modern day Western countries.

#### ***Chemical exposure and its health consequences on the next generation***

In recent years, studies in rodents have established a causal link between *in utero* exposure to endocrine disruptors and the development of kidney and prostate diseases as well as genital tract abnormalities later in adults. Furthermore, the works of Dr Skinner’s research group support that these abnormalities are, in turn, passed down to the males of subsequent generations and are associated with DNA methylation abnormalities and transcriptional alterations [8, 9]. However, the data is still to be confirmed by other independent studies [10, 11].

#### ***Transgenerational non-genetic inheritance of olfactory imprint***

Imprinting sensory inputs present in the environment during infancy is an adaptive behavior that is conserved in every animal species as well as in humans. Imprinting establishes a positive attachment to the native environment; it drives many species-specific behaviors at the adult stages. In the worm, odor stimuli in the young larvae environment leave life-long lasting imprints that enhance attraction of the adults for the imprinted odors [12]. Using this model, the authors were able to demonstrate that imprinting can be transmitted to the following generation. In addition, they showed that small RNAs purified from imprinted worms successfully transferred imprinting to naïve worms by the way of ingestion. Finally, they demonstrated that if the environmental change does not persist (odors removed), inheritance of adaptation is limited to one generation. In contrast, when odors were maintained in the environment during at least five generations, adaptation became fixed and innately expressed in the population. The new phenotype (imprint) is associated with RNA production and is dependent on RNAi machinery and chromatin modification effectors. An efficient system of siRNA amplification ensures the maintenance of the gene silencing even after the initial trigger is gone, allowing siRNA to be transmitted to the progeny [12, 13]. Heritable RNAi defective mutants have been isolated through genetic screens that identified a number of *sid* (systemic interference deficient) genes, including *sid-1*, encoding a non-specific RNA canal, or *hrde-1*, encoding an Argonaute protein [12–14].

#### ***Early stress and its implications in progeny health***

In addition to diet and nutrition, environmental factors encountered in early life can also have an impact on the subsequent generations. In particular, environmental factors may produce trans-generational effects on behavior and brain functions [15–17]. In humans, women exposed to negative behavioral factors such as childhood neglect or abuse are more likely to have children with a higher susceptibility to psychiatric disorders later in life, even when those children are raised in normal conditions where they are not exposed to neglect or abuse [15]. This has pointed to the existence of potential molecular mechanisms that are independent of the environment during pregnancy or the maternal care and which confer disease susceptibility by transmitting older molecular alterations induced by environmental factors.

With the advent of mouse models for early trauma and stress, studies in animals have started addressing the implications of those environment-independent molecular mechanisms. One of the most relevant models is based on unpredictable maternal separation combined with unpredictable maternal stress (MSUS) in the mouse, a manipulation that mimics early chronic stress in humans. When the impact of MSUS on the offspring of the stressed animals was examined, it was found that the manipulation induced multiple behavioral alterations. It resulted in severe symptoms of depression and impulsivity, and in impaired social behaviors in the stressed animals (F1 generation) after they became adults. Furthermore, these behavioral symptoms were also observed to a similar extent in the immediate offspring (F2 generation) of the stressed animals, even though these offspring were reared normally and never exposed to any stress. The following F3 generation also exhibited similar behavioral alterations, indicating that the manipulation had had a persistent effect that could be transmitted across several generations [18–20]. Transmission occurred through both females and males, suggesting the existence of multiple mechanisms for epigenetic transgenerational inheritance. A greater understanding of the molecular bases of the effects of such experiences on the brain and on the germline epigenome should one day aid in the treatment of mental illnesses.

### Transgenerational non-genetic inheritance of newly acquired phenotypes induced by genetic alteration

#### ***Transgenerational epigenetic inheritance of longevity in C. Elegans***

Our environment (i.e., dietary habits, chemical exposure) can positively or negatively impact our lives and the ones of our progeny. A study performed by A. Brunet’s team has demonstrated that our environment can also affect our longevity and that of our descendants. They showed that epigenetic regulators implicated in histone modifications, a type of chromatin modification, could affect the longevity of the worm. More importantly, the lifespan of the progeny was affected even when the original modification was no longer present in the descendants [21].

#### ***Transgenerational epigenetic inheritance in the mouse***

Another example of transgenerational epigenetic heredity was observed at two independent loci in the mouse, the viable yellow *Agouti* locus (*Avy*) [22] and the *Axin* fused locus (*Axin*^*Fu*^) [23]. The phenotype variations observed at these two loci correlated with the methylation status of transposons located upstream of the genes [24]. In *Axin*^*Fu*^, the phenotypes correlated with differential DNA methylation at a retrotransposon within *Axin*^*Fu*^ and the transcription of a mutant close to the retrotransposon LTR. The parental phenotype was transmitted by both the paternal and the maternal alleles for the *Avy* locus, or by the maternal allele solely for the *Axin*^*Fu*^ locus.

### Paramutation: a process of RNA-mediated transgenerational inheritance of new regulatory states

#### ***Paramutation in plants***

In 1956, Alexander Brink defined the paramutation as an interaction between 2 alleles of a gene that causes a heritable modification in gene expression [25]. The paramutation can be defined with 3 key characteristics. First, the new pattern of gene expression is transmitted to subsequent generations, even if the inducing allele is not present. Second, the allele which received the new epigenetic information continues to provide this new information to the other allele. Third, the nucleotidic sequence of the affected allele is not altered, indicating that an epigenetic modification is involved in this process. Although the molecular mechanisms of the paramutation remain to be clarified, an increasing number of data point to RNA molecules as crucial players. In plants, several“paramutable” loci (the expression of which can be altered by paramutations) have been analyzed [26]. In corn, the locus *b1* encoding a transcription factor that drives the biosynthesis of the purple pigment anthocyanin, was the most analyzed. On the one hand, the sequences required for the paramutation, namely the repeated sequences located approximately 100 kb upstream of the promoter, were transcribed and led to the production of non-coding double-stranded RNAs important for the silencing of the targeted loci [27]. This region is differentially methylated and contains a chromatin structure that differs between the wild-type and the paramutated allele. This process is called RNA-directed DNA methylation or RdDM. Importantly, extensive screens of mutants that are deficient either in the establishment or in the maintenance of paramutations have uncovered several RNA-polymerases dependent on RNA (RdRP) that are strictly required for the transcriptional gene inactivation. While the control and the maintenance of the epigenetic states are yet to be fully understood, all of those results converge toward a leading role for RNA molecules.

#### ***Paramutation in the mouse***

The mouse paramutation was first described in the inherited epigenetic modification of expression of the *Kit* gene, which results in a fur-color phenotype convenient for genetic studies. In the homozygous state, *null* mutations of the *Kit* gene (a tyrosine kinase receptor) are lethal because of altered differentiation and migration of the melanoblasts. In contrast, heterozygotes show a characteristic distribution of white fur patches (“white-spotting”). Importantly, we noticed that in the progeny of heterozygote intercrosses and of crosses among wild type partners, the white patch feature was maintained in the homozygous *Kit*^*+/+*^ progeny. The two wild type alleles were structurally normal at the level of the DNA sequence, thus revealing that the molecular modifications occurred at an epigenetic level. Strikingly, similar to the plant paramutation, the mutant phenotype was efficiently inherited in the subsequent generations and biological assays support the hypothesis that RNA molecules might be the transgenerational vectors. Surprisingly, the microinjection into one-cell stage embryos of RNAs extracted from either sperm or somatic organs of heterozygote animals appeared to be efficient in induction of white fur patches phenotype in the mouse. The resulting mice exhibited the mutant *kit* phenotype and, strikingly, the modified phenotype was inherited throughout 3 generations thus demonstrating that RNA molecules injected into fertilized eggs were able to modify epigenetically the expression pattern of a targeted gene. These results are somehow in line with the previously discussed mechanisms of hereditary phenotypic changes inherited upon transfer of RNAs in *Caenorhabditis elegans*[28]. The concept of epigenetic heredity induced by RNA molecules was then extended to the microinjection of various miRs into one-cell stage embryos. For instance, microRNA miR-1 microinjection induced a heritable murine hypertrophic cardiomyopathy. In this model, the paramutation induced the transcriptional activation of *Cdk9*, a known main regulator of cardiac development in humans [29]. The microinjection of miR-124 was another experiment which further supported the role of RNA-mediated epigenetic inheritance in mice [30]. The miR-124 microinjected mice grew a body size 30–40 percent larger than controls and had a frequent occurrence of twin pregnancies. The phenotype was associated with the over expression of the *Sox9* gene during early embryogenesis and, importantly, this epigenetic modification was transmitted over 3 generations. Given that the molecular mechanisms of RNA-mediated heredity have remained largely unknown, we hypothesized that the modifications that are crucial to the transgenerational effect may not be variations in the sequence repertoire. Our recent results took us to the still poorly explored world of RNA covalent modifications. The lead was an absolute requirement for the RNA methyltransferase Dnmt2 for the Kit* and Sox9* paramutations [31]. Unlike the other members of the Dnmt family of DNA methyltransferase, Dnmt2 is an RNA methyltransferase, tRNAs being the first recognized substrates [32], of which methylation protects them against endonucleolytic cleavage [33]. Current speculations and our own recent results suggest a more general role in the maintenance of small noncoding RNAs.

#### ***Paramutation in Drosophila***

In *Drosophila*, the repression of cluster of transposable elements depends on the production of piRNA (small RNAs implicated in PIWI proteins-mediated silencing of transposable elements) and induces strong trans-silencing effect (TSE). Recently, Ronsseray’s team has demonstrated that this cluster can convert other homologous transgene clusters, that are incompetent for TSE, into strong silencers, by conferring a new ability to produce piRNA. The acquired silencing capacity is very stable and can be maintained over 50 generations [34, 35].

In terms of evolution, the conservation across different species of RNA-mediated transgenerational inheritance of modified phenotypes highlights the importance of this process. The molecular mechanisms involved are not well-defined, however all the aforementioned examples (many others exist) provide evidence that RNAs are implicated in several important processes, such as transposon regulation (pi-RNA), heterochromatin formation (RdRP), developmental gene regulation, and genome stability (Table 1). Altogether, these data converge toward the same idea: RNAs can alter gene expression in such a way that the modification can be transmitted to the next generations. Whether this type of inheritance might be at work in human remains a challenging open question.

**Table 1:** **Overview of transgenerational epigenetic inheritance**

Inherited phenotype	Description	Reference
*Caenorhabditis elegans*		
Lifespan	Deficiency in histone H3 Lysine 4 trimethylation complex extends the *C. elegans* longevity and that of its progeny. This occurs though the original modification is no longer present in the descendants and is associated with epigenetic gene expression alteration.	[21]
Olfactory imprinting	Exposure of *C. elegans* to olfactory cues during specific and restricted time windows leaves a permanent epigenetic memory (“olfactory imprint”). The initial trigger is olfactory stimulation of sensory neurons and may involve synthesis and spreading of small RNAs produced from endogenous sources of unknown dsRNA. The olfactory imprinting is restricted to one generation when the environmental change is removed, but becomes fixed if odors are maintained for at least five generations.	[12, 36]
*Drosophila melanogaster*		
Silencing of transposable elements	A cluster of transposable elements that is dependent on the production of small RNAs to repress other homologous transgenes after it acquired the ability to produce piRNA. This molecular alteration is maintained over 50 generations.	[34]
*Mus musculus*		
Metabolic phenotype	Diet induced paternal and/or maternal inheritance of metabolic phenotypes	[6, 7, 37–39]
White-tailed phenotype	Induction of a modified phenotype by microinjection of RNAs into fertilized eggs. The modified phenotype is maintained over 3 generations.	[40]
Overgrowth phenotype		
		[30]
Hypertrophy phenotype		
		[29]
Kinked tail phenotype	Inheritance of an epigenetic state (DNA methylation) at specific loci.	[22, 23]
Fur color phenotype		
Spermatogenesis alteration	Diet and/or drug exposure induced transgenerational inheritance of reproductive alterations.	[8]
*Human*		
Metabolic phenotype	Increased frequency of diabetes was related to scarcity of food to the grandfathers.	[41]
Behavior phenotype and brain dysfunction	Children from mothers exposed to negative behavioral factors have an increased susceptibility to psychiatric disorders.	[15–17]

### Concluding remarks - a role for RNA molecules in the transgenerational paternal inheritance of newly acquired phenotypes

As mentioned above, hereditary transmission of familial traits, including physiological and developmental processes, cannot not always be explained by the Mendelian schemes [1], and we may now consider an alternative, non-exclusive concept, of a heritable epigenetic trait directed and transmitted across generations by epigenetic mechanisms. Thus, in contrast to the general thought that somatic and germ cell epigenetic signatures are fully reprogrammed, it appears that distinct marks can escape the reprogramming and be transgenerationally inherited. The two major epigenetic marks, namely DNA methylation and chromatin structure, are likely to be involved in both maternal and paternal inheritance. Indeed, contrary to the well-established dogma, it has been shown that even in sperm, nucleosomes are retained in the chromatin and might be implicated in epigenetic regulation [42, 43]. In addition, the non-coding RNA might be another player involved in this process. The next question to be addressed is whether non coding RNAs are also implicated in paternal epigenetic inheritance. Indeed, sperm is known to be a transcriptionally inert entity devoid of cytoplasm. Based on this, it is hard to speculate whether spermatozoa RNA might also play a role in paternal epigenetic inheritance. However, it is now well established that several classes of RNA molecules are present in human sperm [44, 45], and comprise a large variety of molecules, generally of small sizes, among which are transcript fragments and microRNAs. Results in *C. elegans, Drosophila*, and mouse strongly support that in addition to opening avenues for the identification of the molecular players involved in male infertility [46, 47], spermatozoa RNA may play important roles in the regulation of early embryonic gene expression and carry transgenerational epigenetic information [48]. According to this current line of thinking, it is tempting to speculate that “spermatozoal RNAs”, once delivered into the oocytes, may serve as a scaffold for the recruitment of chromatin modifying complexes which will induce epigenetic modifications at the targeted locus throughout embryonic development and adult life. Although results strongly suggest that this molecular regulation is at work in mice, it remains to be demonstrated that it is conserved in humans.

## Abbreviations

RNAi: RNA-mediated interference
siRNA: Small Interfering RNA
hrde-1 heritable: RNAi defective 1
miRNA: MicroRNA
Cdk9: Cyclin-dependent kinase 9
Sox9: SRY-box containing gene 9
Dnmt: DNA methyltransferase
Dnmt2: DNA methyltransferase 2
piRNA: Piwi-interacting RNA
LTR: Long terminal repeats
dsRNA: Double-stranded RNA
RdRp: RNA-dependent RNA polymerase
RdDM: RNA-directed DNA methylation
TSE: Trans-silencing Effect
MSUS: Unpredictable maternal separation and maternal stress.
